# Ameloblastoma of the Sinonasal Tract: Report of a Case with Clinicopathologic Considerations

**DOI:** 10.1155/2012/218156

**Published:** 2012-11-28

**Authors:** Maria Grazia Tranchina, Paolo Amico, Antonio Galia, Carmela Emmanuele, Vincenzo Saita, Filippo Fraggetta

**Affiliations:** ^1^Pathology Unit, Cannizzaro Hospital, Via Messina 829, 95126 Catania, Italy; ^2^Umberto I Hospital, Via Trieste 24, 94100 Enna, Italy; ^3^Cervicofacial Surgery Unit, Cannizzaro Hospital, Via Messina 829, 95126 Catania, Italy

## Abstract

Ameloblastomas are locally aggressive jaw tumours with a high propensity for recurrence and are believed to arise from remnants of dental lamina or odontogenic epithelium. Extragnathic ameloblastomas are unusual, and primary sinonasal tract origin is very uncommon with few cases reported in the literature. We herein report a case of primary sinonasal ameloblastoma presented in a 74-year-old male with nasal obstruction, rhinorrhoea, and sinusitis. Nasal endoscopy showed the right nasal cavity completely obstructed by a polypoid lesion attached to the lateral nasal wall. A preoperative CT scan was performed showing a solid lesion, measuring 2 cm in the maximum diameter, extending from the nasopharynx area with obstruction of the ostiomeatal unit and sphenoethmoidal recess into the lateral pharyngeal space, laterally to the parotid, without continuity with maxillary alveola and antrum. The tumour was completely excised endoscopically, and a final diagnosis of ameloblastoma was rendered. At the 12-month followup, there was no evidence of recurrence.

## 1. Introduction

Ameloblastoma (AM) is a common odontogenic epithelial tumor, usually arising in the maxilla or mandible [1, 2]. It probably arises from cell rests of the dental lamina or from the odontogenic epithelium and is characterized by high propensity for recurrence with a locally aggressive behavior [1–6]. Malignant transformation of otherwise ameloblastoma has also been reported [7]. Tumors that grow in the maxilla may secondarily extend through the nasal and paranasal cavities [1], but primary AMs of sinonasal tract, without connection with gnathic areas, are unusual, with few cases reported in the literature [1, 8–22]. We herein report the clinicopathologic features of a primary sinonasal AM in a 74-year-old male presenting with nasal obstruction and sinusitis. Its clinical presentation and rarity can mislead the clinician into incorrect diagnosis and treatment.

## 2. Case Presentation

**Figure 1:**
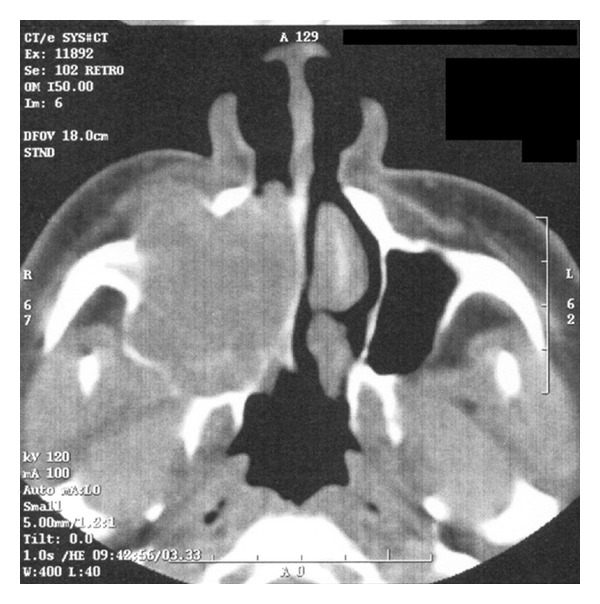
A preoperative CT showing a large lytic expansile, solid lesion extending from the nasopharynx area with obstruction of the ostiomeatal unit, and sphenoethmoidal recess into the lateral pharyngeal space, laterally to the parotid, without continuity with maxillary alveola and antrum.

**Figure 2:**
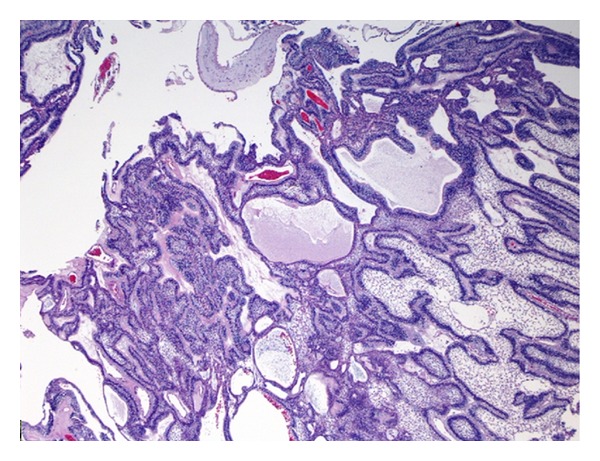
Cords and follicular islands of cytologically benign odontogenic epithelium permeated an edematous, myxoid, hypocellular stroma.

**Figure 3:**
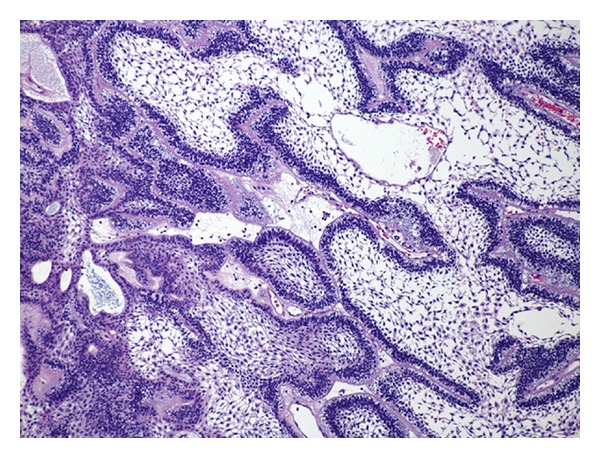
Columnar cells that displayed palisading with classic basaloid (“follicular”) appearance and reverse polarity lined the periphery of the epithelium.

**Figure 4:**
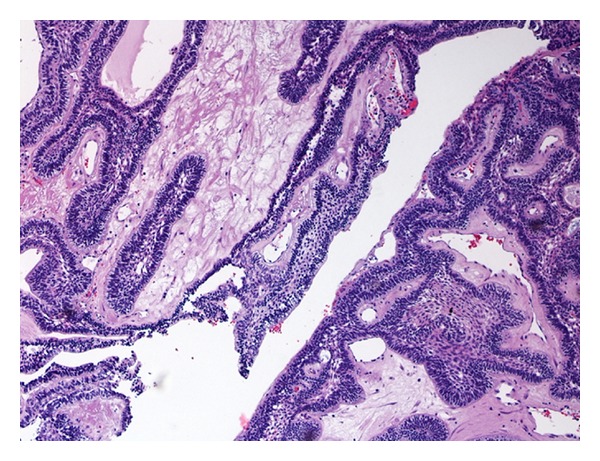
In some areas, stroma was collagenized.

**Figure 5:**
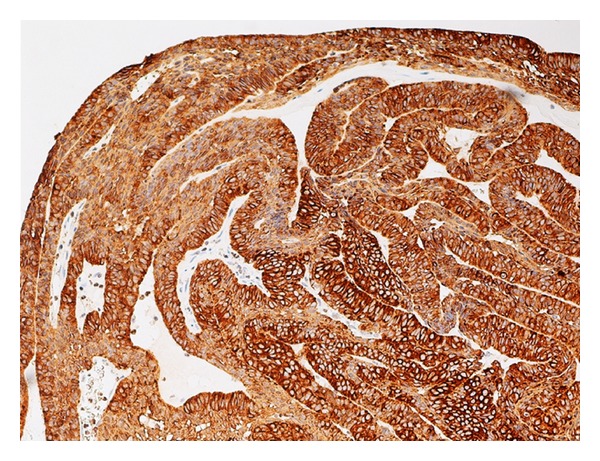
Neoplastic cells showing a diffuse immunostaining with CK AE1/AE3.

A 74-year-old man presented to our hospital with a 2-month history of progressive right-sided nasal obstruction, associated with rhinorrhoea and sinusitis. He had no associated history of epistaxis, pain, or anosmia and was otherwise healthy, with no previous sinonasal disease. Nasal endoscopy showed the right nasal cavity completely obstructed by an exophytic, polypoid, firm lesion, with irregularly shaped borders, attached to the lateral nasal wall. A preoperative CT scan was performed showing a lytic expansile, solid lesion, measuring 2 cm in the maximum diameter extending from the nasopharynx area into the lateral pharyngeal space, laterally to the parotid, without continuity with maxillary alveola and antrum (Figure 1). Neoplasm obstructed the ostiomeatal unit and sphenoethmoidal recess and caused erosion of bone in the middle cranial fossa. The presence of unilateral involvement and bone erosion on CT raised suspicion for a neoplasm, and the mass was excised via endoscopic sinus surgery under general anesthesia. Anterior and posterior nasal packing was done. Haemostasis was achieved. On histologic examination, cords and islands of cytologically benign odontogenic epithelium permeated an edematous, myxoid, hypocellular stroma (Figure 2). Columnar cells that displayed palisading with classic basaloid appearance and reverse polarity lined the periphery of the epithelium (Figure 3). In some areas, stroma was collagenized (Figure 4). Two predominant patterns of growth were seen: follicular and plexiform. Neoplastic cells exhibited strong reactivity for CK AE1/AE3 (Figure 5), CK 5 and 14. Based on the clinicoradiologic and morphoimmunohistochemical findings, a diagnosis of primary sinonasal follicular/plexiform ameloblastoma was rendered. The patient has no sign of recurrence after a 1-year followup.

## 3. Discussion

AM is a slow growing, locally aggressive tumor, usually arising in the jaws and frequently associated with an unerupted tooth [1–10]. It may arise from the epithelial lining of a dentigerous cyst or from the remnants of the dental lamina and enamel organ [5, 7, 20]. AM appears most commonly in the third to fifth decades, but it has also been described in children and adolescence [1, 4, 8, 20]. There is no gender predilection.

Primary sinonasal AMs are rare and have a predilection for men of older ages [1, 2, 9, 20], as in our case. Clinically, sinonasal AMs may present as sinusitis, nasal obstruction, epistaxis, or may be asymptomatic [8–12, 16, 20]. Primary sinonasal AMs may be intraosseous or extraosseous/peripheral [1], as well as those located in the oral cavity. 

As far as histogenetic considerations about sinonasal AM origin is concerned, some studies have indicated a close relation of the embryological derivation of the sinonasal tract and odontogenic apparatus [1, 4, 5, 13–16, 22]. The sinonasal tract and oral cavity communicate, in fact, until closure of the palatine shelves. This proximity during embryological development could explain the ability of the sinonasal tract mucosa either to incorporate the odontogenic epithelium or to acquire cells capable of odontogenesis during development. Accordingly, primary sinonasal AMs probably arise from remnants of odontogenic epithelium, while AMs of oral cavity could arise from remnants of the dental lamina within the gingival or from surface epithelium that has retained the capacity to differentiate along odontogenic structures. It is, in fact, most often found in the soft tissues of the posterior gingival [1, 4, 5]. It has been also suggested that peripheral gnathic and sinonasal AM may originate from pluripotential stem cells of the basal layer of the oral and sinonasal epithelium, respectively [4, 5, 7, 20].

From a pathological viewpoint, AM has been divided into solid and (multi)cystic types [1, 2, 5], but this distinction is often arbitrary since nearly all AMs show some degree of cystic change. AM is generally composed of nests and cords of ameloblastic epithelium separated by relatively small amounts of fibrous connective-tissue stroma. Follicular and plexiform are the most predominant pattern [1, 2]. In the follicular subtype, the epithelial islands contain central portions composed of a loose network resembling that of the enamel organ. The epithelium at the periphery is composed of tall, columnar cells with hyperchromatic nuclei. In the plexiform subtype, the epithelium is arranged in interconnecting strands and cords of epithelium in loose, vascular, sparsely cellular connective tissue stroma. The epithelial cells are in close juxtaposition and appear basaloid or cuboidal. Other histologic subtypes may also occur, often in the same tumour, including basal cell, granular cell, desmoplastic, and acanthomatous types [1, 2].

Sinonasal AMs are often indistinguishable from benign nasal polyps or chronic sinusitis both clinically and on CT scan. Although the presence of unilateral involvement and bony erosion on CT should raise suspicion for a neoplasm, because of the lack of pathognomonic radiologic characteristics, a definitive diagnosis of sinonasal AM requires biopsy. However, the differential diagnosis may remain a challenge in small biopsies if tissue fragments obtained for diagnosis are superficial, and the typical histology of the tumor is not well represented in them.

The overwhelming majority of cases affecting the sinonasal cavity are tumors that grow in the maxilla and secondarily extend through the nasal and paranasal cavities. Therefore, of primary importance is to exclude extension into the sinonasal tract from a primary gnathic AM. 

 AM histologic features are pathognomonic; therefore, the differential diagnosis is limited. It comprises acute and chronic sinusitis, inverted papilloma, carcinoma, adenocarcinoma, angiofibroma nasopharyngeal, and basal cell adenoma, a rare salivary gland-type tumour primitive of sinonasal tract [1–4, 23]. The differential diagnosis could also include sinonasal extension of a craniopharyngioma, the nature of which should be apparent on MRI or CT scan examinations [4, 24].

AM is a lesion of low-grade malignant potential, histologically benign but locally aggressive with a marked tendency for recurrence. So, AM requires complete excision with adequate margins to minimize recurrence. Surgical excision is the treatment of choice in all cases. Recently, endoscopic management of AMs has resulted in less radical surgical approach with decreased morbidity and better tumor control [8–10, 12, 14, 16–19]. However, prognosis is based on the extension of the lesion and on adjacent structures involved rather than on origin of the lesion. Simple curettage is associated with unacceptable recurrence rates. Recurrence often presents after 15 years or more, and it is important to emphasize the need for long-term periodic followup [1–4, 8, 9, 17–19].
